# Crystal structures of di­bromido­{*N*-[(pyridin-2-yl-κ*N*)methyl­idene]picolinohydrazide-κ^2^ *N*′,*O*}cadmium methanol monosolvate and di­iodido{*N*-[(pyridin-2-yl-κ*N*)methyl­idene]picolinohydrazide-κ^2^ *N*′,*O*}cadmium

**DOI:** 10.1107/S2056989017005308

**Published:** 2017-04-13

**Authors:** Ali Akbar Khandar, Farhad Akbari Afkhami, Harald Krautscheid, Kenneth Aase Kristoffersen, Zeliha Atioğlu, Mehmet Akkurt, Carl Henrik Görbitz

**Affiliations:** a Young Researchers and Elite Club, Tabriz Branch, Islamic Azad University, Tabriz, Iran; b Universität Leipzig, Fakultätfür Chemie und Mineralogie, Johannisallee 29, D-04103 Leipzig, Germany; c Department of Chemistry, University of Oslo, PO Box 1033 Blindern, N-0315 Oslo, Norway; d İlke Education and Health Foundation, Cappadocia Vocational College, The Medical Imaging Techniques Program, 50420 Mustafapaşa, Ürgüp, Nevşehir, Turkey; e Department of Physics, Faculty of Sciences, Erciyes University, 38039 Kayseri, Turkey

**Keywords:** crystal structure, hydrazone: tridentate ligand, cadmium, bromide, iodide, hydrogen bonding

## Abstract

The title compounds, [CdBr_2_(C_12_H_10_N_4_O)]·CH_3_OH, (I), and [CdI_2_(C_12_H_10_N_4_O)], (II), are cadmium bromide and cadmium iodide complexes of the ligand (*E*)-*N*′-(pyridin-2-yl­methyl­ene)picolinohydrazide. Complex (I) crystallizes as the methanol monosolvate. In both compounds, the Cd^2+^ cation is ligated by one O atom and two N atoms of the tridentate ligand, and by two bromide anions forming a Br_2_N_2_O penta­coordination sphere for (I), and by two iodide anions forming an I_2_N_2_O penta­coordination sphere for (II), both with a distorted square-pyramidal geometry. In the crystal of complex (I), mol­ecules are linked by pairs of N—H⋯O and O—H⋯Br hydrogen bonds, involving the solvent mol­ecule, forming dimeric units, which are linked by C—H⋯Br hydrogen bonds forming layers parallel to (101). In the crystal of complex (II), mol­ecules are linked by N—H⋯I hydrogen bonds, forming chains propagating along [010]. In complex (II), measured at room temperature, the two iodide anions are each disordered over two sites; the refined occupancy ratio is 0.75 (2):0.25 (2).

## Abstract 2

The title compounds are cadmium bromide and cadmium iodide complexes of the ligand *N*′-(pyridin-2-yl­methyl­ene)picolinohydrazide. In both compounds, the Cd^2+^ ion is ligated by one O atom and two N atoms of the tridentate ligand, and by two halide ions. Both have fivefold coordination spheres with a distorted square-pyramidal geometry.

## Chemical context   

The cadmium(II) ion, has a *d* ^10^ electronic configuration and exhibits a variety of coordination geometries and modes. Hydrazone ligands are one of the most important classes of flexible and versatile polydentate ligands and show very high efficiency in chelating transition metal ions (Afkhami *et al.*, 2017*a* ▸). Hydrazone ligands obtained from 2-pyridine carb­oxy­lic acid can act as ditopic ligands *via* two different donor sites (a tridentate coordination pocket and through an N-donor pyridine group), and have the potential to form mono- and multinuclear structures (Afkhami *et al.*, 2017*b* ▸). Herein, we report on the crystal structures of two new Cd^II^ complexes based on the tridentate hydrazone ligand, (*E*)-*N*′-(pyridin-2-yl­methyl­ene)picolinohydrazide, obtained by condensation of an equimolar mixture of 2-pyridine­carbaldehyde and picolinic acid hydrazide in methanol.

## Structural commentary   

**Figure 1:**
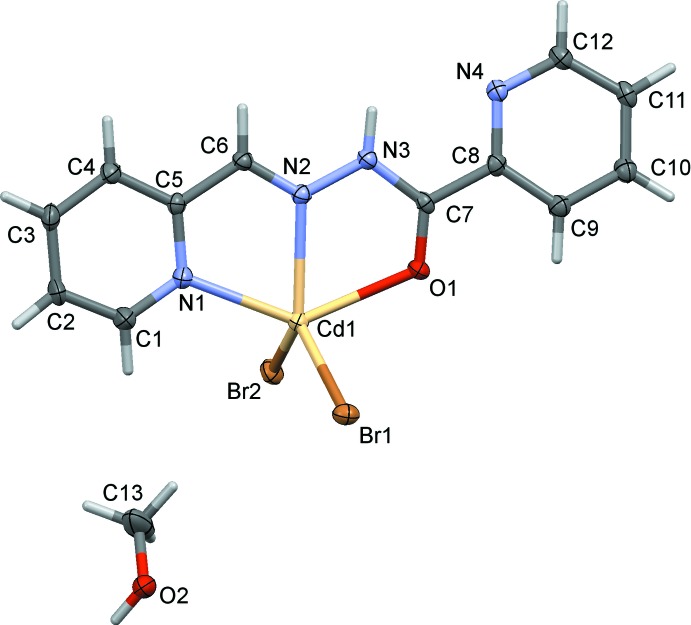
The mol­ecular components in the structure of compound (I), with atom labelling. Displacement ellipsoids are shown at the 30% probability level.

The mol­ecular structures of compounds (I) and (II) are shown in Figs. 1 ▸ and 2 ▸, respectively. In compound (I), the ligand is almost planar with a dihedral angle between the pyridine rings of 6.9 (3)°. The Cd1—Br1 and Cd1—Br2 bond lengths are 2.5585 (6) and 2.5490 (7) Å, respectively, and the Cd1—N2 bond length is 2.336 (4) Å. Atom Cd1 is ligated by one O atom (O1) and two N atoms (N1 and N2) of the tridentate ligand, and by two bromide anions, hence the Cd^2+^ cation has a fivefold Br_2_N_2_O coordination sphere with a distorted shape and a τ_5_ value of 0.33 (τ_5_ = 0 for an ideal square-pyramidal coordination sphere, and = 1 for an ideal trigonal-pyramidal coordination sphere; Addison *et al.*, 1984 ▸).
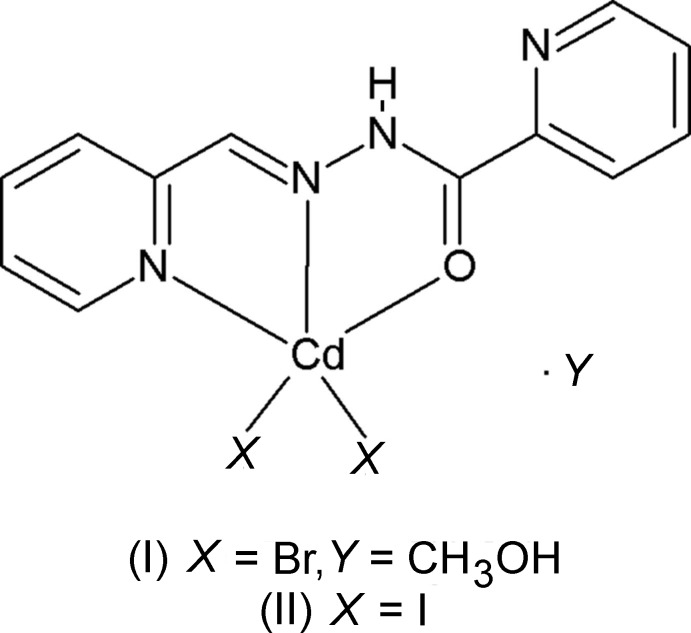


In compound (II), the ligand is also almost planar with a dihedral angle between the pyridine rings of 8.0 (2)°. The two iodide anions are each disordered over two sites; the refined occupancy ratio is 0.75 (2):0.25 (2) for atoms I1*A*/I2*A*:I1*B*/I2*B*. Considering the major components only, the Cd1—I1*A* and Cd1—I2*A* bond lengths are 2.736 (3) and 2.7128 (19) Å, respectively, and the Cd1—N2 bond length is 2.336 (3) Å. Atom Cd1 is ligated by one O atom (O1) and two N atoms (N1 and N2) of the tridentate ligand, and by two iodide anions. Atom Cd1 has a fivefold I_2_N_2_O coordination sphere with a distorted shape and a τ_5_ value of 0.28.

## Supra­molecular features   

**Figure 3:**
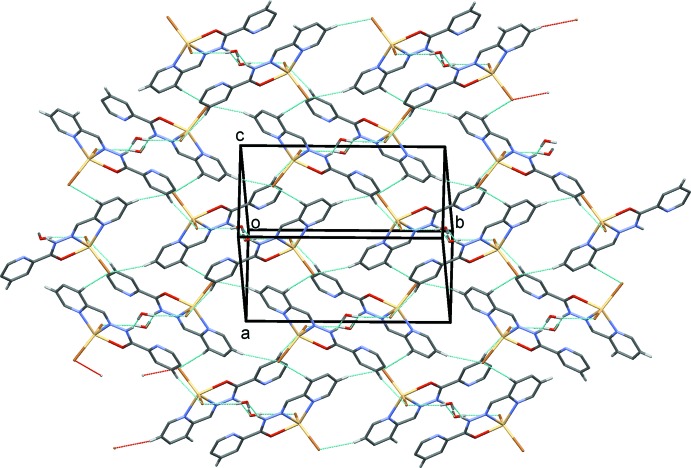
A view normal to (101) of the crystal packing of compound (I). The hydrogen bonds are shown as dashed lines (see Table 1 ▸). For clarity, only the H atoms involved in hydrogen bonding have been included.

**Figure 4:**
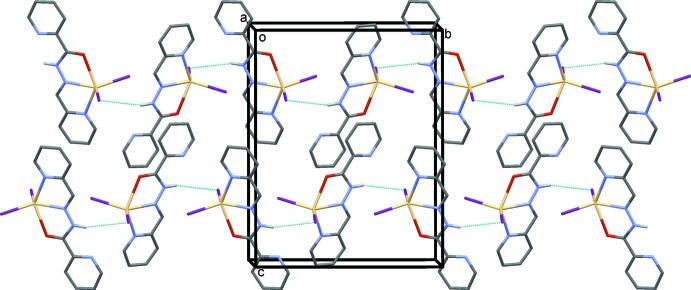
A view along the *a* axis of the crystal packing of compound (II). The hydrogen bonds are shown as dashed lines (see Table 2 ▸). For clarity, only the H atoms involved in hydrogen bonding and only the major components of the disordered I atoms have been included.

In the crystal of compound (I), mol­ecules are linked by pairs of N—H⋯O and O—H⋯Br hydrogen bonds, involving the solvent mol­ecule, forming dimeric units, which are linked by C—H⋯Br hydrogen bonds forming layers parallel to (101); see Table 1 ▸ and Fig. 3 ▸. In the crystal of complex (II), mol­ecules are linked by N—H⋯I hydrogen bonds forming chains propagating along [010]; see Table 2 ▸ and Fig. 4 ▸.

## Database survey   

All bond lengths and angles in the title compounds fall within acceptable ranges and are comparable with those reported for related structures, such as bis­{*N*′-[(*E*)-4-hy­droxy­benzyl­idene]pyridine-4-carbohydrazide-κ*N* ^1^}di­iodido­cadmium methanol disolvate (Afkhami *et al.*, 2017*c* ▸), di­bromido­{*N*′-[1-(pyridin-2-yl)ethyl­idene]picolinohydrazide-*κ* ^2^ *N*′,*O*}cadmium (Akkurt *et al.*, 2012 ▸), di-μ-chlorido-bis­(chlorido­{*N*′-[phenyl-(pyridin-2-yl-κ*N*)methyl­idene]pyridine-2-carbohydrazide-κ^2^ *N*′,*O*}cadmium) (Akkurt *et al.*, 2014 ▸), bis­{2-[(2,4-di­methyl­phen­yl)imino­meth­yl]pyridine-κ^2^ *N*,*N′*}bis­(thio­cyanato-κ*N*)cadmium (Malekshah­ian *et al.*, 2012 ▸), and *cis*-di­aqua­bis-[(*E*)-4-(2-hy­droxybenzyl­idene­amino)­benzoato-κ^2^ *O*,*O′*]cadmium in which layers are built from strong O—H⋯O hydrogen bonds (Yao *et al.*, 2006 ▸).

## Synthesis and crystallization   

A solution of the ligand *N*′-(pyridin-2-yl­methyl­ene)picolinohydrazide (0.151 g, 0.5 mmol) in 30 ml of methanol was treated with a methano­lic solution of the appropriate cadmium(II) salt (0.5 mmol); CdBr_2_ for complex (I) and CdI_2_ for (II). The solutions were heated under reflux for 4 h and then allowed to stand at room temperature. After slow evaporation of the solvent, single crystals separated out. They were collected, washed with ether and dried over P_4_O_10_ in vacuum.

## Refinement   

Crystal data, data collection and structure refinement details for compounds (I) and (II) are summarized in Table 3 ▸. For complex (I), measured at 130 K, H atoms were placed in calculated positions (C—H = 0.95–0.98 Å, N—H = 0.88 Å and O—H = 0.84 Å) and included in the refinement in the riding-model approximation, with *U*_iso_(H) = 1.5*U*_eq_(O) and 1.2*U*_eq_(N,C) for other H atoms. Owing to poor agreement, two reflections, (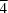 4 6 and 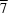 10 3), were omitted from the final cycles of refinement. For complex (II), measured at 296 K, the C-bound H atoms were placed in calculated positions (C—H = 0.93 Å) and included in the refinement in the riding-model approximation, with *U_i_*_so_(H) = 1.2*U*_eq_(C). The N-bound H atoms were located in a difference-Fourier map but were refined with a distance restraint of N—H = 0.86 (4) Å with *U*_iso_(H) = 1.2*U*_eq_(N). In complex (II), the two iodide anions (I1 and I2) are each disordered over two sites, and their site-occupation factors refined to 0.75 (2):0.25 (2).

## Supplementary Material

Crystal structure: contains datablock(s) I, II, global. DOI: 10.1107/S2056989017005308/su5361sup1.cif

Structure factors: contains datablock(s) I. DOI: 10.1107/S2056989017005308/su5361Isup2.hkl

Structure factors: contains datablock(s) II. DOI: 10.1107/S2056989017005308/su5361IIsup3.hkl

CCDC references: 1543006, 1543005

Additional supporting information:  crystallographic information; 3D view; checkCIF report

## Figures and Tables

**Figure 2:**
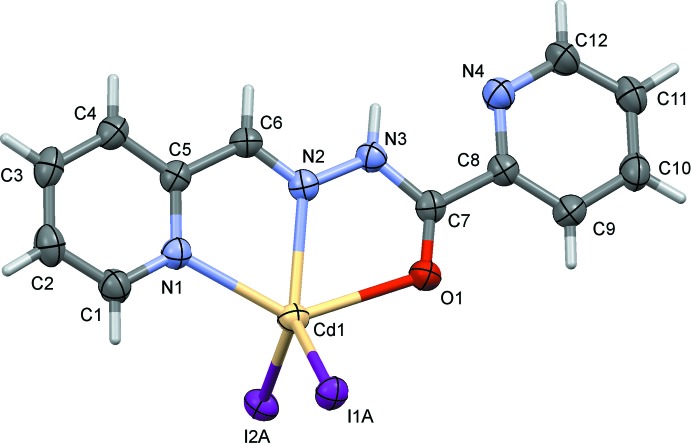
The mol­ecular structure of compound (II), with atom labelling. Displacement ellipsoids are shown at the 30% probability level. Only the major components of the disordered I atoms are shown.

**Table 1:** Hydrogen-bond geometry (Å, °) for (I)

*D*—H⋯*A*	*D*—H	H⋯*A*	*D*⋯*A*	*D*—H⋯*A*
N3—H3*N*⋯O2^i^	0.88	1.96	2.803 (5)	161
O2—H2*A*⋯Br1^ii^	0.84	2.70	3.456 (4)	150
C2—H2⋯Br2^iii^	0.95	2.90	3.734 (6)	147
C4—H4⋯Br2^iv^	0.95	2.91	3.826 (5)	162
C10—H10⋯Br1^v^	0.95	2.85	3.703 (5)	149

Symmetry codes: (i) 
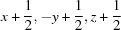
; (ii) 
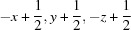
; (iii) 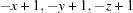; (iv) 
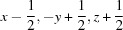
; (v) 
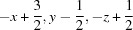
.

**Table 2:** Hydrogen-bond geometry (Å, °) for (II)

*D*—H⋯*A*	*D*—H	H⋯*A*	*D*⋯*A*	*D*—H⋯*A*
N3—H3*N*⋯I2*A* ^i^	0.87 (4)	3.04 (4)	3.866 (3)	161 (3)

Symmetry code: (i) 
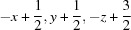
.

**Table 3:** Experimental details

	(I)	(II)
Crystal data		
Chemical formula	[CdBr_2_(C_12_H_10_N_4_O)]·CH_4_O	[CdI_2_(C_12_H_10_N_4_O)]
*M*_r_	530.50	592.44
Crystal system, space group	Monoclinic, *P*2_1_/*n*	Monoclinic, *P*2_1_/*n*
Temperature (K)	130	296
*a*, *b*, *c* (Å)	7.5482 (4), 15.7571 (8), 14.6407 (6)	7.5264 (7), 13.1325 (12), 16.5718 (15)
β (°)	95.132 (4)	94.384 (1)
*V* (Å^3^)	1734.35 (15)	1633.2 (3)
*Z*	4	4
Radiation type	Cu *K*α	Mo *K*α
μ (mm^−1^)	15.59	5.12
Crystal size (mm)	0.08 × 0.05 × 0.04	0.48 × 0.20 × 0.02

Data collection		
Diffractometer	Agilent SuperNova, Dual, Cu at zero, Atlas	Bruker D8 Venture diffractometer with Photon 100 CMOS detector
Absorption correction	Multi-scan (*CrysAlis PRO*; Agilent, 2011 ▸)	Multi-scan (*SADABS*; Bruker, 2016 ▸)
*T*_min_, *T*_max_	0.561, 1.000	0.616, 0.903
No. of measured, independent and observed [*I* > 2σ(*I*)] reflections	6773, 3458, 2764	3946, 3946, 3193
*R*_int_	0.038	0.023
(sin θ/λ)_max_ (Å^−1^)	0.625	0.665

Refinement		
*R*[*F* ^2^ > 2σ(*F* ^2^)], *wR*(*F* ^2^), *S*	0.034, 0.078, 1.02	0.023, 0.058, 1.06
No. of reflections	3458	3946
No. of parameters	201	204
H-atom treatment	H-atom parameters constrained	H atoms treated by a mixture of independent and constrained refinement
Δρ_max_, Δρ_min_ (e Å^−3^)	0.63, −0.76	0.65, −0.39

Computer programs: *CrysAlis PRO* (Agilent, 2011 ▸), *APEX3* and *SAINT-Plus* (Bruker, 2016 ▸), *SHELXS2014/7* (Sheldrick, 2008 ▸), *SHELXT* (Sheldrick, 2015*a* ▸), *SHELXL2016/6* and *SHELXL2014* (Sheldrick, 2015*b* ▸), *ORTEP-3 for Windows* and *WinGX* (Farrugia, 2012 ▸), *Mercury* (Macrae *et al.*, 2008 ▸) and *PLATON* (Spek, 2009 ▸).

## supplementary crystallographic information

### (I) Dibromido{N-[(pyridin-2-yl-κN)methylidene]picolinohydrazide-κ2N',O}cadmium methanol monosolvate Crystal data

**Table:**

[CdBr_2_(C_12_H_10_N_4_O)]·CH_4_O	*F*(000) = 1016
*M**_r_* = 530.50	*D*_x_ = 2.032 Mg m^−^^3^
Monoclinic, *P*2_1_/*n*	Cu *K*α radiation, λ = 1.5418 Å
*a* = 7.5482 (4) Å	Cell parameters from 2435 reflections
*b* = 15.7571 (8) Å	θ = 3.0–74.4°
*c* = 14.6407 (6) Å	µ = 15.59 mm^−^^1^
β = 95.132 (4)°	*T* = 130 K
*V* = 1734.35 (15) Å^3^	Prism, light yellow
*Z* = 4	0.08 × 0.05 × 0.04 mm

### (I) Dibromido{N-[(pyridin-2-yl-κN)methylidene]picolinohydrazide-κ2N',O}cadmium methanol monosolvate Data collection

**Table:**

Agilent SuperNova, Dual, Cu at zero, Atlas diffractometer	3458 independent reflections
Radiation source: SuperNova (Cu) X-ray Source	2764 reflections with *I* > 2σ(*I*)
Mirror monochromator	*R*_int_ = 0.038
ω scans	θ_max_ = 74.6°, θ_min_ = 4.1°
Absorption correction: multi-scan (CrysAlis PRO; Agilent, 2011)	*h* = −9→9
*T*_min_ = 0.561, *T*_max_ = 1.000	*k* = −18→19
6773 measured reflections	*l* = −18→10

### (I) Dibromido{N-[(pyridin-2-yl-κN)methylidene]picolinohydrazide-κ2N',O}cadmium methanol monosolvate Refinement

**Table:**

Refinement on *F*^2^	0 restraints
Least-squares matrix: full	Hydrogen site location: inferred from neighbouring sites
*R*[*F*^2^ > 2σ(*F*^2^)] = 0.034	H-atom parameters constrained
*wR*(*F*^2^) = 0.078	*w* = 1/[σ^2^(*F*_o_^2^) + (0.0275*P*)^2^] where *P* = (*F*_o_^2^ + 2*F*_c_^2^)/3
*S* = 1.02	(Δ/σ)_max_ = 0.001
3458 reflections	Δρ_max_ = 0.63 e Å^−^^3^
201 parameters	Δρ_min_ = −0.76 e Å^−^^3^

### (I) Dibromido{N-[(pyridin-2-yl-κN)methylidene]picolinohydrazide-κ2N',O}cadmium methanol monosolvate Special details

**Table:**

Geometry. All esds (except the esd in the dihedral angle between two l.s. planes) are estimated using the full covariance matrix. The cell esds are taken into account individually in the estimation of esds in distances, angles and torsion angles; correlations between esds in cell parameters are only used when they are defined by crystal symmetry. An approximate (isotropic) treatment of cell esds is used for estimating esds involving l.s. planes.

### (I) Dibromido{N-[(pyridin-2-yl-κN)methylidene]picolinohydrazide-κ2N',O}cadmium methanol monosolvate Fractional atomic coordinates and isotropic or equivalent isotropic displacement parameters (Å^2^)

**Table:**

	*x*	*y*	*z*	*U*_iso_*/*U*_eq_	
C1	0.3542 (9)	0.3896 (4)	0.5136 (4)	0.0494 (16)	
H1	0.3566	0.4180	0.4565	0.059*	
C2	0.2666 (9)	0.4292 (4)	0.5819 (4)	0.0482 (16)	
H2	0.2058	0.4814	0.5708	0.058*	
C3	0.2712 (8)	0.3898 (4)	0.6662 (3)	0.0376 (12)	
H3	0.2154	0.4151	0.7150	0.045*	
C4	0.3584 (7)	0.3128 (3)	0.6786 (3)	0.0315 (11)	
H4	0.3643	0.2850	0.7364	0.038*	
C5	0.4375 (6)	0.2763 (3)	0.6056 (3)	0.0259 (10)	
C6	0.5190 (6)	0.1927 (3)	0.6130 (3)	0.0257 (9)	
H6	0.5270	0.1620	0.6690	0.031*	
C7	0.7080 (6)	0.0589 (3)	0.4588 (3)	0.0239 (9)	
C8	0.7879 (6)	−0.0271 (3)	0.4567 (3)	0.0261 (10)	
C9	0.8393 (6)	−0.0581 (3)	0.3740 (3)	0.0311 (11)	
H9	0.8205	−0.0258	0.3192	0.037*	
C10	0.9185 (7)	−0.1374 (4)	0.3737 (3)	0.0338 (11)	
H10	0.9544	−0.1609	0.3185	0.041*	
C11	0.9440 (6)	−0.1815 (3)	0.4546 (3)	0.0306 (11)	
H11	1.0005	−0.2354	0.4566	0.037*	
C12	0.8857 (7)	−0.1459 (3)	0.5335 (3)	0.0321 (11)	
H12	0.9033	−0.1772	0.5890	0.039*	
C13	0.2406 (12)	0.4864 (5)	0.2809 (5)	0.083 (3)	
H13A	0.3593	0.5002	0.2631	0.124*	
H13B	0.2374	0.4268	0.2999	0.124*	
H13C	0.2133	0.5229	0.3321	0.124*	
N1	0.4340 (6)	0.3153 (3)	0.5234 (3)	0.0333 (10)	
N2	0.5791 (5)	0.1625 (3)	0.5414 (2)	0.0240 (8)	
N3	0.6507 (5)	0.0828 (3)	0.5400 (2)	0.0265 (8)	
H3N	0.6589	0.0495	0.5884	0.032*	
N4	0.8071 (5)	−0.0709 (3)	0.5358 (2)	0.0267 (8)	
O1	0.6971 (5)	0.1057 (2)	0.3907 (2)	0.0291 (7)	
O2	0.1154 (6)	0.4997 (3)	0.2070 (2)	0.0401 (9)	
H2A	0.1202	0.5505	0.1898	0.060*	
Cd1	0.57044 (5)	0.24361 (2)	0.40754 (2)	0.02814 (10)	
Br1	0.34115 (7)	0.21728 (4)	0.27099 (3)	0.03309 (13)	
Br2	0.82753 (8)	0.34184 (4)	0.37807 (4)	0.03634 (14)	

### (I) Dibromido{N-[(pyridin-2-yl-κN)methylidene]picolinohydrazide-κ2N',O}cadmium methanol monosolvate Atomic displacement parameters (Å^2^)

**Table:**

	*U*^11^	*U*^22^	*U*^33^	*U*^12^	*U*^13^	*U*^23^
C1	0.081 (5)	0.035 (4)	0.033 (3)	0.020 (3)	0.011 (3)	0.004 (2)
C2	0.076 (4)	0.028 (3)	0.041 (3)	0.024 (3)	0.012 (3)	−0.003 (2)
C3	0.049 (3)	0.027 (3)	0.038 (3)	0.003 (2)	0.009 (2)	−0.011 (2)
C4	0.042 (3)	0.024 (3)	0.029 (2)	−0.004 (2)	0.006 (2)	−0.001 (2)
C5	0.031 (2)	0.021 (3)	0.026 (2)	−0.003 (2)	0.0001 (17)	−0.0046 (18)
C6	0.031 (2)	0.022 (3)	0.024 (2)	−0.003 (2)	0.0019 (17)	0.0018 (18)
C7	0.022 (2)	0.017 (2)	0.032 (2)	−0.0017 (18)	−0.0005 (17)	−0.0006 (19)
C8	0.026 (2)	0.023 (3)	0.029 (2)	−0.0033 (19)	−0.0015 (17)	−0.0036 (19)
C9	0.031 (2)	0.032 (3)	0.030 (2)	0.003 (2)	0.0019 (19)	−0.001 (2)
C10	0.034 (3)	0.030 (3)	0.039 (3)	0.002 (2)	0.008 (2)	−0.001 (2)
C11	0.027 (2)	0.020 (3)	0.045 (3)	0.0005 (19)	0.003 (2)	−0.002 (2)
C12	0.031 (2)	0.025 (3)	0.039 (2)	0.000 (2)	0.001 (2)	0.003 (2)
C13	0.123 (8)	0.050 (5)	0.065 (4)	0.023 (5)	−0.045 (5)	−0.006 (4)
N1	0.050 (3)	0.025 (2)	0.0260 (18)	0.004 (2)	0.0066 (17)	−0.0026 (17)
N2	0.0269 (19)	0.023 (2)	0.0222 (16)	−0.0018 (16)	0.0007 (14)	−0.0002 (15)
N3	0.033 (2)	0.021 (2)	0.0258 (18)	0.0001 (17)	0.0004 (15)	0.0017 (16)
N4	0.0272 (19)	0.024 (2)	0.0295 (18)	0.0008 (17)	0.0035 (15)	−0.0002 (16)
O1	0.0388 (19)	0.0215 (19)	0.0274 (16)	0.0022 (15)	0.0046 (13)	0.0044 (14)
O2	0.060 (2)	0.032 (2)	0.0284 (17)	0.0022 (19)	0.0041 (16)	−0.0001 (15)
Cd1	0.03855 (18)	0.0225 (2)	0.02370 (15)	0.00179 (15)	0.00451 (12)	0.00180 (13)
Br1	0.0344 (3)	0.0331 (3)	0.0313 (2)	−0.0030 (2)	0.00060 (19)	0.0069 (2)
Br2	0.0439 (3)	0.0243 (3)	0.0413 (3)	−0.0036 (2)	0.0063 (2)	−0.0008 (2)

### (I) Dibromido{N-[(pyridin-2-yl-κN)methylidene]picolinohydrazide-κ2N',O}cadmium methanol monosolvate Geometric parameters (Å, º)

**Table:**

C1—N1	1.319 (7)	C9—H9	0.9500
C1—C2	1.394 (7)	C10—C11	1.370 (7)
C1—H1	0.9500	C10—H10	0.9500
C2—C3	1.378 (8)	C11—C12	1.391 (7)
C2—H2	0.9500	C11—H11	0.9500
C3—C4	1.385 (8)	C12—N4	1.324 (7)
C3—H3	0.9500	C12—H12	0.9500
C4—C5	1.395 (6)	C13—O2	1.388 (7)
C4—H4	0.9500	C13—H13A	0.9800
C5—N1	1.349 (6)	C13—H13B	0.9800
C5—C6	1.453 (7)	C13—H13C	0.9800
C6—N2	1.271 (6)	N1—Cd1	2.351 (4)
C6—H6	0.9500	N2—N3	1.368 (6)
C7—O1	1.236 (5)	N2—Cd1	2.336 (4)
C7—N3	1.354 (6)	N3—H3N	0.8800
C7—C8	1.485 (7)	O1—Cd1	2.396 (3)
C8—N4	1.344 (6)	O2—H2A	0.8400
C8—C9	1.393 (6)	Cd1—Br2	2.5490 (7)
C9—C10	1.385 (7)	Cd1—Br1	2.5585 (6)
			
N1—C1—C2	124.1 (5)	C12—C11—H11	120.6
N1—C1—H1	118.0	N4—C12—C11	123.9 (5)
C2—C1—H1	118.0	N4—C12—H12	118.1
C3—C2—C1	117.7 (5)	C11—C12—H12	118.1
C3—C2—H2	121.1	O2—C13—H13A	109.5
C1—C2—H2	121.1	O2—C13—H13B	109.5
C2—C3—C4	119.1 (5)	H13A—C13—H13B	109.5
C2—C3—H3	120.5	O2—C13—H13C	109.5
C4—C3—H3	120.5	H13A—C13—H13C	109.5
C3—C4—C5	119.4 (5)	H13B—C13—H13C	109.5
C3—C4—H4	120.3	C1—N1—C5	118.3 (4)
C5—C4—H4	120.3	C1—N1—Cd1	125.0 (3)
N1—C5—C4	121.3 (5)	C5—N1—Cd1	116.7 (3)
N1—C5—C6	117.0 (4)	C6—N2—N3	121.8 (4)
C4—C5—C6	121.6 (4)	C6—N2—Cd1	120.1 (3)
N2—C6—C5	117.3 (4)	N3—N2—Cd1	118.1 (3)
N2—C6—H6	121.4	C7—N3—N2	115.3 (4)
C5—C6—H6	121.4	C7—N3—H3N	122.4
O1—C7—N3	122.6 (4)	N2—N3—H3N	122.4
O1—C7—C8	121.7 (4)	C12—N4—C8	116.7 (4)
N3—C7—C8	115.7 (4)	C7—O1—Cd1	117.1 (3)
N4—C8—C9	123.5 (5)	C13—O2—H2A	109.5
N4—C8—C7	117.6 (4)	N2—Cd1—N1	68.80 (14)
C9—C8—C7	118.9 (4)	N2—Cd1—O1	66.95 (12)
C10—C9—C8	118.2 (5)	N1—Cd1—O1	135.60 (13)
C10—C9—H9	120.9	N2—Cd1—Br2	120.65 (9)
C8—C9—H9	120.9	N1—Cd1—Br2	102.70 (12)
C11—C10—C9	118.8 (5)	O1—Cd1—Br2	102.51 (8)
C11—C10—H10	120.6	N2—Cd1—Br1	122.22 (9)
C9—C10—H10	120.6	N1—Cd1—Br1	109.42 (11)
C10—C11—C12	118.9 (5)	O1—Cd1—Br1	91.20 (8)
C10—C11—H11	120.6	Br2—Cd1—Br1	115.99 (2)
			
N1—C1—C2—C3	3.2 (11)	C2—C1—N1—Cd1	176.8 (5)
C1—C2—C3—C4	−1.3 (10)	C4—C5—N1—C1	0.4 (8)
C2—C3—C4—C5	−0.8 (8)	C6—C5—N1—C1	177.2 (5)
C3—C4—C5—N1	1.4 (8)	C4—C5—N1—Cd1	−179.2 (4)
C3—C4—C5—C6	−175.4 (5)	C6—C5—N1—Cd1	−2.3 (6)
N1—C5—C6—N2	−0.6 (7)	C5—C6—N2—N3	−176.9 (4)
C4—C5—C6—N2	176.2 (5)	C5—C6—N2—Cd1	3.4 (6)
O1—C7—C8—N4	174.9 (4)	O1—C7—N3—N2	0.2 (6)
N3—C7—C8—N4	−4.3 (6)	C8—C7—N3—N2	179.3 (4)
O1—C7—C8—C9	−5.0 (7)	C6—N2—N3—C7	179.5 (4)
N3—C7—C8—C9	175.9 (4)	Cd1—N2—N3—C7	−0.8 (5)
N4—C8—C9—C10	−1.7 (7)	C11—C12—N4—C8	−1.6 (7)
C7—C8—C9—C10	178.1 (4)	C9—C8—N4—C12	2.7 (7)
C8—C9—C10—C11	−0.5 (7)	C7—C8—N4—C12	−177.1 (4)
C9—C10—C11—C12	1.5 (7)	N3—C7—O1—Cd1	0.5 (6)
C10—C11—C12—N4	−0.5 (8)	C8—C7—O1—Cd1	−178.6 (3)
C2—C1—N1—C5	−2.7 (10)		

### (I) Dibromido{N-[(pyridin-2-yl-κN)methylidene]picolinohydrazide-κ2N',O}cadmium methanol monosolvate Hydrogen-bond geometry (Å, º)

**Table:**

*D*—H···*A*	*D*—H	H···*A*	*D*···*A*	*D*—H···*A*
N3—H3*N*···O2^i^	0.88	1.96	2.803 (5)	161
O2—H2*A*···Br1^ii^	0.84	2.70	3.456 (4)	150
C2—H2···Br2^iii^	0.95	2.90	3.734 (6)	147
C4—H4···Br2^iv^	0.95	2.91	3.826 (5)	162
C10—H10···Br1^v^	0.95	2.85	3.703 (5)	149

Symmetry codes: (i) *x*+1/2, −*y*+1/2, *z*+1/2; (ii) −*x*+1/2, *y*+1/2, −*z*+1/2; (iii) −*x*+1, −*y*+1, −*z*+1; (iv) *x*−1/2, −*y*+1/2, *z*+1/2; (v) −*x*+3/2, *y*−1/2, −*z*+1/2.

### (II) Diiodido{N-[(pyridin-2-yl-κN)methylidene]picolinohydrazide-κ2N',O}cadmium Crystal data

**Table:**

[CdI_2_(C_12_H_10_N_4_O)]	*F*(000) = 1088
*M**_r_* = 592.44	*D*_x_ = 2.409 Mg m^−^^3^
Monoclinic, *P*2_1_/*n*	Mo *K*α radiation, λ = 0.71073 Å
*a* = 7.5264 (7) Å	Cell parameters from 6468 reflections
*b* = 13.1325 (12) Å	θ = 2.5–28.0°
*c* = 16.5718 (15) Å	µ = 5.12 mm^−^^1^
β = 94.384 (1)°	*T* = 296 K
*V* = 1633.2 (3) Å^3^	Plate, light yellow
*Z* = 4	0.48 × 0.20 × 0.02 mm

### (II) Diiodido{N-[(pyridin-2-yl-κN)methylidene]picolinohydrazide-κ2N',O}cadmium Data collection

**Table:**

Bruker D8 Venture diffractometer with Photon 100 CMOS detector	3946 independent reflections
Radiation source: fine-focus sealed tube	3193 reflections with *I* > 2σ(*I*)
Graphite monochromator	*R*_int_ = 0.023
Detector resolution: 8.3 pixels mm^-1^	θ_max_ = 28.2°, θ_min_ = 2.0°
Sets of exposures each taken over 0.5° ω rotation scans	*h* = −9→10
Absorption correction: multi-scan (SADABS; Bruker, 2016)	*k* = −17→17
*T*_min_ = 0.616, *T*_max_ = 0.903	*l* = −21→21
3946 measured reflections	

### (II) Diiodido{N-[(pyridin-2-yl-κN)methylidene]picolinohydrazide-κ2N',O}cadmium Refinement

**Table:**

Refinement on *F*^2^	0 restraints
Least-squares matrix: full	Hydrogen site location: mixed
*R*[*F*^2^ > 2σ(*F*^2^)] = 0.023	H atoms treated by a mixture of independent and constrained refinement
*wR*(*F*^2^) = 0.058	*w* = 1/[σ^2^(*F*_o_^2^) + (0.0256*P*)^2^ + 0.6207*P*] where *P* = (*F*_o_^2^ + 2*F*_c_^2^)/3
*S* = 1.06	(Δ/σ)_max_ = 0.001
3946 reflections	Δρ_max_ = 0.65 e Å^−^^3^
204 parameters	Δρ_min_ = −0.39 e Å^−^^3^

### (II) Diiodido{N-[(pyridin-2-yl-κN)methylidene]picolinohydrazide-κ2N',O}cadmium Special details

**Table:**

Geometry. All esds (except the esd in the dihedral angle between two l.s. planes) are estimated using the full covariance matrix. The cell esds are taken into account individually in the estimation of esds in distances, angles and torsion angles; correlations between esds in cell parameters are only used when they are defined by crystal symmetry. An approximate (isotropic) treatment of cell esds is used for estimating esds involving l.s. planes.

### (II) Diiodido{N-[(pyridin-2-yl-κN)methylidene]picolinohydrazide-κ2N',O}cadmium Fractional atomic coordinates and isotropic or equivalent isotropic displacement parameters (Å^2^)

**Table:**

	*x*	*y*	*z*	*U*_iso_*/*U*_eq_	Occ. (<1)
C1	0.5553 (5)	0.3164 (3)	0.9481 (2)	0.0622 (9)	
H1	0.5027	0.2525	0.9506	0.075*	
C2	0.6695 (5)	0.3481 (3)	1.0130 (2)	0.0691 (10)	
H2	0.6933	0.3064	1.0578	0.083*	
C3	0.7462 (5)	0.4426 (3)	1.0092 (2)	0.0662 (9)	
H3	0.8220	0.4664	1.0520	0.079*	
C4	0.7095 (4)	0.5017 (3)	0.9414 (2)	0.0591 (8)	
H4	0.7609	0.5657	0.9376	0.071*	
C5	0.5948 (4)	0.4646 (2)	0.87896 (18)	0.0481 (7)	
C6	0.5559 (4)	0.5222 (2)	0.80398 (18)	0.0503 (7)	
H6	0.5999	0.5879	0.7986	0.060*	
C7	0.3276 (4)	0.4766 (2)	0.61592 (18)	0.0488 (7)	
C8	0.2989 (4)	0.5342 (2)	0.53845 (18)	0.0498 (7)	
C9	0.2074 (5)	0.4925 (3)	0.4719 (2)	0.0635 (9)	
H9	0.1609	0.4269	0.4732	0.076*	
C10	0.1865 (5)	0.5520 (3)	0.4020 (2)	0.0695 (10)	
H10	0.1230	0.5271	0.3558	0.083*	
C11	0.2590 (5)	0.6460 (3)	0.4019 (2)	0.0692 (10)	
H11	0.2461	0.6865	0.3558	0.083*	
C12	0.3516 (6)	0.6809 (3)	0.4704 (2)	0.0727 (11)	
H12	0.4031	0.7453	0.4695	0.101 (15)*	
N1	0.5174 (3)	0.3727 (2)	0.88258 (15)	0.0496 (6)	
N2	0.4609 (3)	0.48064 (19)	0.74667 (15)	0.0473 (6)	
N3	0.4243 (4)	0.5284 (2)	0.67483 (16)	0.0508 (6)	
H3N	0.461 (5)	0.590 (3)	0.668 (2)	0.061*	
N4	0.3717 (5)	0.6265 (2)	0.53900 (18)	0.0659 (8)	
O1	0.2696 (3)	0.39087 (18)	0.62576 (14)	0.0592 (6)	
Cd1	0.32992 (3)	0.32196 (2)	0.76532 (2)	0.05080 (8)	
I1A	0.4779 (3)	0.1408 (2)	0.7239 (2)	0.0549 (3)	0.75 (2)
I2A	−0.0093 (3)	0.31832 (13)	0.81000 (15)	0.0555 (4)	0.75 (2)
I1B	0.4749 (10)	0.1451 (7)	0.7364 (9)	0.0654 (16)	0.25 (2)
I2B	0.0012 (11)	0.3249 (6)	0.8180 (7)	0.0787 (18)	0.25 (2)

### (II) Diiodido{N-[(pyridin-2-yl-κN)methylidene]picolinohydrazide-κ2N',O}cadmium Atomic displacement parameters (Å^2^)

**Table:**

	*U*^11^	*U*^22^	*U*^33^	*U*^12^	*U*^13^	*U*^23^
C1	0.071 (2)	0.060 (2)	0.055 (2)	0.0027 (17)	0.0032 (17)	0.0053 (16)
C2	0.073 (2)	0.081 (3)	0.053 (2)	0.018 (2)	−0.0004 (17)	0.0083 (18)
C3	0.058 (2)	0.088 (3)	0.0519 (19)	0.0085 (19)	−0.0055 (15)	−0.0109 (18)
C4	0.0529 (18)	0.065 (2)	0.0587 (19)	−0.0021 (16)	0.0016 (15)	−0.0105 (16)
C5	0.0423 (15)	0.0520 (18)	0.0500 (16)	0.0055 (13)	0.0039 (12)	−0.0044 (13)
C6	0.0490 (17)	0.0468 (18)	0.0551 (18)	−0.0015 (13)	0.0048 (13)	−0.0003 (14)
C7	0.0480 (16)	0.0503 (18)	0.0485 (16)	0.0069 (13)	0.0062 (13)	−0.0016 (13)
C8	0.0491 (17)	0.0503 (17)	0.0502 (17)	0.0049 (13)	0.0059 (13)	0.0020 (13)
C9	0.067 (2)	0.065 (2)	0.058 (2)	−0.0120 (17)	0.0011 (16)	−0.0026 (17)
C10	0.071 (2)	0.088 (3)	0.0490 (19)	−0.004 (2)	−0.0041 (16)	0.0038 (18)
C11	0.075 (2)	0.077 (3)	0.055 (2)	0.006 (2)	0.0017 (17)	0.0150 (18)
C12	0.096 (3)	0.058 (2)	0.063 (2)	−0.003 (2)	−0.001 (2)	0.0137 (18)
N1	0.0526 (15)	0.0482 (15)	0.0478 (14)	0.0044 (11)	0.0029 (11)	−0.0017 (11)
N2	0.0469 (14)	0.0479 (15)	0.0473 (13)	0.0069 (11)	0.0043 (11)	0.0008 (11)
N3	0.0601 (16)	0.0426 (14)	0.0494 (14)	0.0027 (12)	0.0028 (12)	0.0037 (11)
N4	0.088 (2)	0.0536 (18)	0.0547 (17)	−0.0011 (15)	−0.0024 (15)	0.0027 (13)
O1	0.0695 (15)	0.0508 (13)	0.0565 (13)	−0.0077 (11)	−0.0013 (11)	0.0046 (10)
Cd1	0.05478 (14)	0.04400 (13)	0.05349 (14)	−0.00171 (9)	0.00328 (10)	−0.00142 (9)
I1A	0.0610 (5)	0.0468 (4)	0.0568 (7)	0.0081 (3)	0.0034 (5)	−0.0055 (4)
I2A	0.0516 (5)	0.0512 (7)	0.0641 (5)	0.0071 (3)	0.0069 (4)	0.0103 (3)
I1B	0.0736 (17)	0.0486 (12)	0.077 (4)	0.0009 (11)	0.0232 (18)	−0.0098 (15)
I2B	0.065 (2)	0.097 (3)	0.077 (2)	0.0126 (15)	0.0233 (17)	0.0117 (15)

### (II) Diiodido{N-[(pyridin-2-yl-κN)methylidene]picolinohydrazide-κ2N',O}cadmium Geometric parameters (Å, º)

**Table:**

C1—N1	1.326 (4)	C9—C10	1.396 (5)
C1—C2	1.389 (5)	C9—H9	0.9300
C1—H1	0.9300	C10—C11	1.350 (6)
C2—C3	1.372 (6)	C10—H10	0.9300
C2—H2	0.9300	C11—C12	1.365 (6)
C3—C4	1.376 (5)	C11—H11	0.9300
C3—H3	0.9300	C12—N4	1.341 (5)
C4—C5	1.384 (5)	C12—H12	0.9300
C4—H4	0.9300	N1—Cd1	2.407 (3)
C5—N1	1.343 (4)	N2—N3	1.355 (4)
C5—C6	1.465 (4)	N2—Cd1	2.336 (3)
C6—N2	1.268 (4)	N3—H3N	0.86 (4)
C6—H6	0.9300	O1—Cd1	2.493 (2)
C7—O1	1.223 (4)	Cd1—I1B	2.626 (10)
C7—N3	1.355 (4)	Cd1—I2B	2.687 (6)
C7—C8	1.492 (4)	Cd1—I2A	2.7128 (19)
C8—N4	1.329 (4)	Cd1—I1A	2.736 (3)
C8—C9	1.370 (5)		
			
N1—C1—C2	123.2 (4)	C12—C11—H11	120.4
N1—C1—H1	118.4	N4—C12—C11	122.9 (4)
C2—C1—H1	118.4	N4—C12—H12	118.6
C3—C2—C1	118.3 (4)	C11—C12—H12	118.6
C3—C2—H2	120.8	C1—N1—C5	118.1 (3)
C1—C2—H2	120.8	C1—N1—Cd1	125.6 (2)
C2—C3—C4	119.2 (3)	C5—N1—Cd1	116.3 (2)
C2—C3—H3	120.4	C6—N2—N3	121.6 (3)
C4—C3—H3	120.4	C6—N2—Cd1	120.3 (2)
C3—C4—C5	119.1 (4)	N3—N2—Cd1	118.0 (2)
C3—C4—H4	120.5	N2—N3—C7	117.6 (3)
C5—C4—H4	120.5	N2—N3—H3N	120 (2)
N1—C5—C4	122.1 (3)	C7—N3—H3N	122 (2)
N1—C5—C6	116.3 (3)	C8—N4—C12	117.4 (3)
C4—C5—C6	121.6 (3)	C7—O1—Cd1	114.6 (2)
N2—C6—C5	118.5 (3)	N2—Cd1—N1	68.43 (9)
N2—C6—H6	120.7	N2—Cd1—O1	66.56 (8)
C5—C6—H6	120.7	N1—Cd1—O1	134.63 (8)
O1—C7—N3	122.9 (3)	N2—Cd1—I1B	125.4 (2)
O1—C7—C8	123.5 (3)	N1—Cd1—I1B	99.6 (3)
N3—C7—C8	113.6 (3)	O1—Cd1—I1B	101.6 (3)
N4—C8—C9	123.5 (3)	N2—Cd1—I2B	116.02 (18)
N4—C8—C7	115.1 (3)	N1—Cd1—I2B	103.4 (3)
C9—C8—C7	121.4 (3)	O1—Cd1—I2B	100.9 (2)
C8—C9—C10	117.5 (3)	I1B—Cd1—I2B	118.5 (2)
C8—C9—H9	121.3	N2—Cd1—I2A	117.87 (7)
C10—C9—H9	121.3	N1—Cd1—I2A	106.89 (9)
C11—C10—C9	119.5 (4)	O1—Cd1—I2A	98.69 (7)
C11—C10—H10	120.2	N2—Cd1—I1A	123.94 (9)
C9—C10—H10	120.2	N1—Cd1—I1A	102.63 (9)
C10—C11—C12	119.2 (4)	O1—Cd1—I1A	97.56 (9)
C10—C11—H11	120.4	I2A—Cd1—I1A	117.57 (7)

### (II) Diiodido{N-[(pyridin-2-yl-κN)methylidene]picolinohydrazide-κ2N',O}cadmium Hydrogen-bond geometry (Å, º)

**Table:**

*D*—H···*A*	*D*—H	H···*A*	*D*···*A*	*D*—H···*A*
N3—H3*N*···I2*A*^i^	0.87 (4)	3.04 (4)	3.866 (3)	161 (3)

Symmetry code: (i) −*x*+1/2, *y*+1/2, −*z*+3/2.
